# A Low-Cost Flexible Optoelectronic Synapse Based on ZnO Nanowires for Neuromorphic Computing

**DOI:** 10.3390/s24237788

**Published:** 2024-12-05

**Authors:** Yongqing Yue, Zixia Yu, Fangpei Li, Wenbo Peng, Quanzhe Zhu, Yongning He

**Affiliations:** 1 School of Microelectronics, Xi’an Jiaotong University, Xi’an 710049, China; 2 The Key Lab of Micro-Nano Electronics and System Integration of Xi’an City, Xi’an 710049, China; 3 Shaanxi Advanced Semiconductor Technology Center Co., Ltd., Xi’an 710077, China

**Keywords:** ZnO, flexible optoelectronic synapse, synaptic plasticity, photoconductivity

## Abstract

Neuromorphic computing, inspired by the brain, holds significant promise for advancing artificial intelligence. Artificial optoelectronic synapses, which can convert optical signals into electrical signals, play a crucial role in neuromorphic computing. In this study, we successfully fabricated a flexible artificial optoelectronic synapse device based on the ZnO/PDMS structure by utilizing the magnetron sputtering technique to deposit the ZnO film on a flexible substrate. Under UV light illumination, the device exhibits excellent synaptic plasticity, including excitatory postsynaptic current (EPSC), short-term potentiation (STP), and paired-pulse facilitation (PPF). By growing ZnO nanowires, we improved the fabrication processes and further enhanced the synaptic properties of the device, demonstrating long-term potentiation (LTP) and the transition from short-term memory (STM) to long-term memory (LTM). Additionally, the device exhibits outstanding flexibility, maintaining stable synaptic plasticity under bending conditions. This device shows broad application potential in mimicking visual systems and is expected to contribute significantly to the development of neuromorphic computing.

## 1. Introduction

With artificial intelligence rapidly advancing, there is an increasing demand for processing large volumes of data, enhancing efficiency, and reducing energy consumption [1]. However, in traditional von Neumann computing systems, data storage and processing units are physically separate, which often results in increased energy consumption, reduced computational capability, and additional hardware costs [2,3]. Furthermore, most current machine learning systems rely on complementary metal oxide semiconductor (CMOS) transistors [4], which are approaching their miniaturization limits, potentially rendering Moore’s Law inapplicable to future technological developments [5]. At present, neuromorphic computing systems inspired by the human brain are gaining widespread attention, which aim to overcome the limitations of von Neumann computing systems [1,5,6,7]. The human brain, with its extremely complex structure comprising approximately 10^11^ neurons and 10^15^ synapses, can process vast amounts of information efficiently and accurately with very low power consumption [8,9,10,11]. Therefore, designing artificial synaptic devices that mimic the structures and functions of biological synapses is an effective approach for advancing neuromorphic computing.

The visual system plays a crucial role in biological perception and information acquisition, as over 80% of external information acquired by organisms is derived from optical stimuli [3,12,13]. Additionally, photons possess the characteristics of high efficiency and low latency [14]. Given this importance, by mimicking the working principles of the eye, artificial optoelectronic synaptic devices can convert optical signals into electrical signals, thereby enabling the perception and understanding of external environments [15]. Moreover, artificial optoelectronic synaptic devices possess features such as non-contact writing, low power consumption, low cross-talk, and high bandwidth, making them more suitable for ultra-high-speed neuromorphic computing [16,17,18]. In recent years, a variety of materials including metal oxides [19], two-dimensional materials [20], organic materials [21], and inorganic metal halide perovskites [22] have been widely used to fabricate artificial optoelectronic synaptic devices. In this research field, Yuan et al. fabricated an optoelectronic synaptic device based on the LIG/α-GaOx MSM structure by modulating the oxygen vacancy concentration in α-GaOx through Ar plasma treatment. This approach enhanced the device’s plasticity, achieving up to 94% classification accuracy on the Mnist handwritten dataset, demonstrating excellent image classification ability and the advantage of sense-storage integration [23]. Additionally, Lin et al. developed a CeO_2_/MoS_2_ heterojunction optoelectronic memristor, achieving tunable artificial optoelectronic synaptic functions. This device demonstrated a multifunctional artificial visual system with electrical storage, light sense and memory, and visual nociceptors [24]. These research findings provide new ideas for the development of artificial optoelectronic synaptic devices.

Metal oxides are widely regarded as ideal materials for fabricating artificial optoelectronic synaptic devices, with ZnO being highly valued for its notable application potential [25]. ZnO is a wide-bandgap semiconductor with a bandgap of 3.37 eV [26] and a thermodynamically stable wurtzite crystal structure [27,28]. Due to the presence of oxygen vacancies, ZnO exhibits excellent electronic properties, particularly persistent photoconductivity (PPC) under ultraviolet (UV) light illumination, which is crucial for fabricating optical-controlled artificial synaptic devices [29,30,31,32]. Currently, the magnetron sputtering process for ZnO is relatively mature [33], and the prepared ZnO thin films exhibit high conductivity, along with good transparency and compactness.

Most reported artificial synaptic devices are typically fabricated on rigid substrates [34]. However, rigid artificial synaptic devices are often challenging to fit with curved, soft skin and may fracture under low strain (approximately 1%) [35], which could limit their practical applications. To address this challenge, researchers have recently begun exploring the fabrication of flexible artificial synaptic devices, aiming to achieve good flexibility and biocompatibility [36] to better accommodate the actual conditions of the human body. Recent advances indicate that these devices can integrate optoelectronic functions to achieve synaptic behavior, such as image recognition and storage [12], nociceptor [37], and wearable applications [38]. However, there are still several bottleneck issues that need to be addressed, including the mechanical instability of materials during deformation and the efficient integration of optoelectronic synapses into wearable devices.

In this study, we proposed and fabricated a flexible artificial optoelectronic synaptic device based on the ZnO/PDMS structure. Compared to traditional complex manufacturing processes, we utilized RF magnetron sputtering technique to deposit the ZnO thin film on the flexible PDMS substrate. Subsequently, to further enhance synaptic properties, ZnO nanowires were grown on the ZnO thin film using the hydrothermal method, which was simple and low-cost. The fabricated device exhibits excellent synaptic plasticity, capable of mimicking various biological synaptic functions, including excitatory postsynaptic current (EPSC), short-term potentiation (STP), long-term potentiation (LTP), transition from short-term memory (STM) to long-term memory (LTM), and paired-pulse facilitation (PPF). Additionally, the device exhibits outstanding flexibility, maintaining stable LTP characteristics under various bending conditions with different curvature radii. The artificial optoelectronic synaptic device developed in this study shows broad application prospects in flexible neuromorphic computing and artificial intelligence.

## 2. Results and Discussion

**Figure 1:**
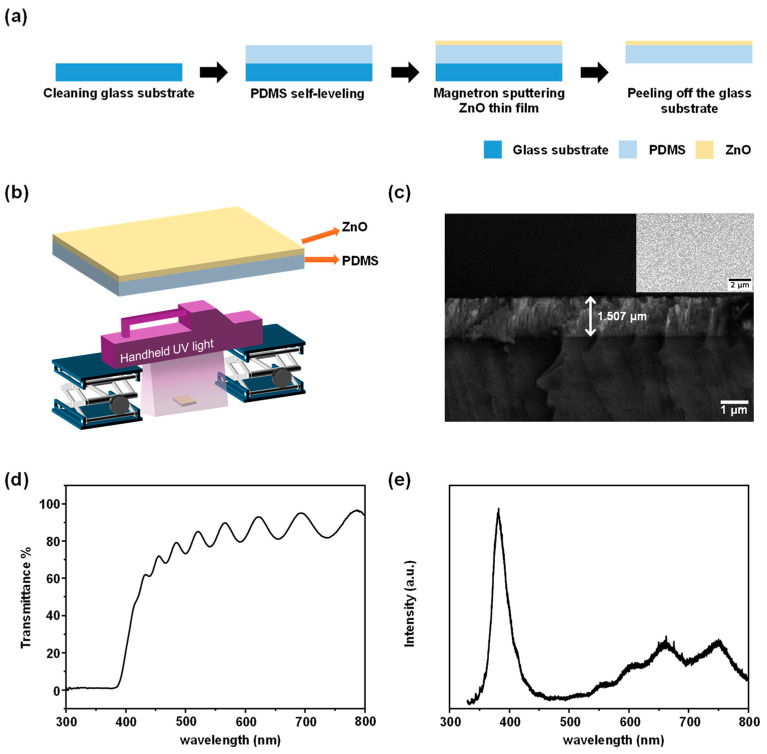
PDMS flexible substrate ZnO artificial optoelectronic synapse device. (**a**) Main fabrication processes. (**b**) Device structure and testing platform. (**c**) SEM images of the ZnO thin film, including the side view and top view (upper right). (**d**) Transmission spectrum. (**e**) PL spectrum.

The main fabrication processes of the device are illustrated in Figure 1a. First, the soda-lime glass substrate undergoes ultrasonic cleaning sequentially with acetone, anhydrous ethanol, and deionized water to remove surface impurity ions and ensure cleanliness. Next, a mixture of PDMS base polymer and curing agent (Shanghai Deji Trading Co., Ltd., Shanghai, China) in a ratio of 5:1 is thoroughly stirred and then placed in a vacuum chamber to eliminate surface bubbles. The bubble-free PDMS is then dropped onto the glass substrate, allowing it to self-level and ensuring uniform distribution on the glass. After this step, the PDMS-covered glass substrate is placed on a heating platform and heated at 120 °C for 10 min to cure the PDMS. Subsequently, the ZnO thin film is deposited on the PDMS surface via magnetron sputtering (High-Vacuum Magnetron Sputtering System, GJP-450, Institute of Microelectronics of the Chinese Academy of Sciences, Beijing, China). The sputtering parameters are Ar flow rate of 10 sccm, pressure of 1.0 Pa, power of 120 W, and sputtering duration of 150 min. Finally, the glass substrate at the bottom of the device is peeled off to obtain the desired PDMS flexible substrate ZnO artificial optoelectronic synapse device. Figure 1b illustrates the schematic diagram of the fabricated device along with the testing platform constructed to explore the device’s response under UV light illumination. The device is placed at the bottom, with the UV light source (wavelength of 365 nm) (UV Irradiation Meter, UV-A, Beijing Normal University Optoelectronic Instrument Factory, Beijing, China) positioned on two adjustable stages. By adjusting the height of the stages, we can modulate the intensity of the UV light, allowing the device to be illuminated under varying intensities for corresponding testing and analysis (Digital Source Meter, B2900A, Keysight Technologies, Santa Rosa, CA, USA).

Figure 1c shows the scanning electron microscopy (SEM) (Field Emission Scanning Electron Microscope, MAIA3 LMH, TESCAN, Brno, Czech Republic) images of the side and top views of the ZnO thin film deposited via magnetron sputtering. The surface of the ZnO thin film exhibits a smooth and dense morphology with a uniform thickness averaging approximately 1.507 μm. To further analyze the chemical composition of the ZnO thin film, X-ray diffraction (XRD) (X-ray Diffractometer, Bruker D8 ADVANCE, Xi’an Langrun International Trade Co., Ltd., Xi’an, China) characterization was performed on the film deposited on a Si substrate, with results illustrated in Figure S1. The XRD patterns indicate that the ZnO thin film exhibits a c-axis orientation, with the characteristic peak at 34.4° corresponding to the (002) crystal plane of ZnO, and the peak at 72° corresponding to the (004) crystal plane. However, due to the high intensity of the characteristic peak of the Si substrate at around 69°, coupled with the relatively low intensity of the (004) peak of ZnO, the (004) peak of ZnO is not prominently visible in the figure. Figure 1d illustrates the transmission spectrum (UV-Visible-NIR Spectrophotometer, PE Lambda950, Seer Network Co., Ltd., Beijing, China) of the ZnO thin film. The ZnO thin film demonstrates a transmission rate of approximately 80% in the visible region, with a sharp decline in transmission around 380 nm in the UV region, attributed to the absorption band edge of ZnO. As shown in Figure 1e, the photoluminescence (PL) spectrum (Raman Spectrometer, Laser Raman Spectrometer, Xi’an Langrun International Trade Co., Ltd., Xi’an, China) of the ZnO thin film was also collected. The emission peak around 380 nm corresponds to the band-edge emission of ZnO, while the small and broad emission peaks observed in the visible region are attributed to deep-level defects in ZnO, primarily associated with oxygen vacancies [39].

**Figure 2:**
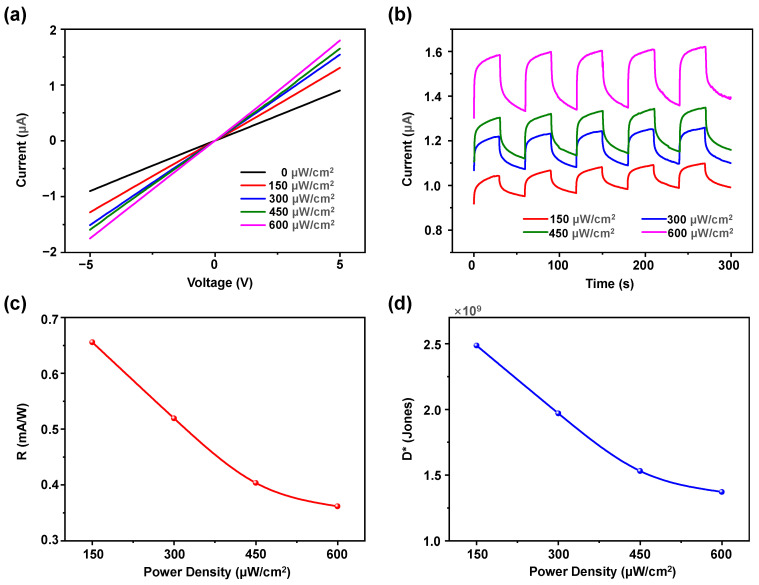
(**a**) I–V characteristics under varying UV light intensities. (**b**) Transient response curves under varying UV light intensities. (**c**) Responsivity (*R*) and (**d**) detectivity (D*) with a bias of +5 V under varying UV light power densities.

Figure 2a illustrates the current–voltage (I–V) characteristics of the device within a voltage sweep range of −5 V to +5 V under dark field conditions and varying UV light intensities. Under dark field conditions, the device exhibits minimal current. When exposed to UV light illumination, the current increased with the UV light intensity. This phenomenon occurs due to the increased number of photogenerated carriers (electron-hole pairs) in ZnO with higher UV light intensities, thereby enhancing the conductivity of the device.

Based on the characteristics of biological synapses, neurons generate action potentials in response to external stimuli, transmitting them to the presynaptic membrane. Upon receiving these signals, the presynaptic membrane releases neurotransmitters that act on the postsynaptic membrane, triggering either excitatory postsynaptic current (EPSC) or inhibitory postsynaptic current (IPSC) [40]. Synaptic plasticity refers to changes in synaptic connections between neurons following external stimuli, manifested as alterations in synaptic weights, which are crucial for the brain’s learning and memory capabilities [38]. In this study, by applying a forward bias, the device will generate an EPSC under UV light illumination, serving as a significant indicator of synaptic weight changes. Depending on the duration of EPSC retention, synaptic plasticity can be categorized into long-term potentiation (LTP) and short-term potentiation (STP), which correspond to long-term memory (LTM) and short-term memory (STM). Figure 2b illustrates the transient response curves of the device under varying UV light intensities with a fixed +5 V bias. The device was exposed to UV light illumination for 30 s followed by 30 s under dark field conditions for each cycle, with five cycles of transient response curves measured for each UV light intensity. It can be observed that following UV light illumination, the device generates an EPSC. After the same duration under dark field conditions, the current does not return to its initial state but remains slightly higher, exhibiting STP characteristics with a degree of non-volatility. The maximum EPSC for each cycle is slightly higher than that of the previous cycle, indicating an increasing trend. Additionally, at the same time point, the EPSC increases with the UV light intensity, which is related to the increase in conductivity caused by higher light intensities.

The sensitivity of the artificial optoelectronic synaptic device to light can be quantified by responsivity (*R*) and detectivity (D*), which are crucial parameters for assessing the optoelectronic performance of the device [41,42]. The responsivity is calculated using the formula *R* = (*I*_photo_ − *I*_dark_)/(*p* × *S*), and the detectivity is given by D* = *R*/(2*q* × *I*_dark_/*S*)^0.5^, where *I*_photo_ is the current under UV light illumination, *I*_dark_ is the current under dark field conditions, *p* is the UV light power density, *S* is the effective area of the device, and *q* is the charge of an electron. Figure 2c,d illustrate the responsivity and detectivity of the device with a +5 V bias under varying UV light power densities. The responsivity ranges from 10 to 1 mA/W, while the detectivity reaches 10^9^ Jones, indicating excellent sensitivity and optoelectronic performance of the artificial optoelectronic synaptic device under UV light illumination. Notably, both responsivity and detectivity exhibit similar trends, decreasing with increasing UV light power density. This phenomenon can be attributed to the limited rates of generation and diffusion of photogenerated carriers as UV light intensity increased, which grow at a slower rate than the power density and may eventually saturate. Consequently, although the photocurrent increases with light intensity, the responsivity and detectivity decreased.

**Figure 3:**
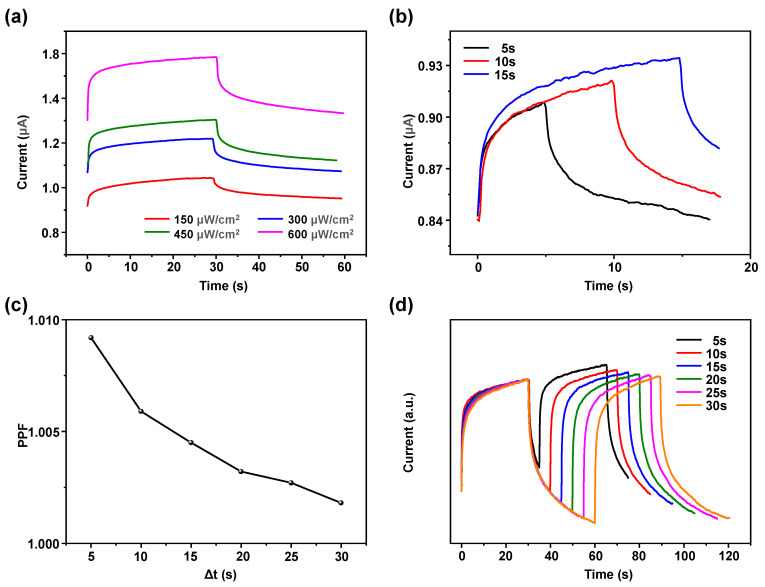
(**a**) Switching characteristics under varying UV light intensities. (**b**) Transient response curves for different illumination durations at the same UV light intensity. (**c**) The PPF variation with varying illumination Δt. (**d**) I–t curves corresponding to each Δt.

Figure 3a illustrates the switching characteristics of the device under varying UV light intensities. The rate of EPSC increase escalates with the rise in UV light intensity for the same illumination duration. This increase is attributed to the generation of more photogenerated carriers within the ZnO semiconductor as the UV light intensity rises, thereby accelerating the current growth rate. This phenomenon is analogous to the brain’s learning process, where stronger stimuli lead to deeper impressions. The greater the intensity of the stimulus, the more profound the memory effect, which in turn results in a faster EPSC growth rate. Additionally, the duration of illumination also influences the EPSC growth rate. Figure 3b illustrates the response curves of the device exposed to 5 s, 10 s, and 15 s of UV light illumination at the same intensity. The results indicate that, at a constant UV light intensity, the EPSC increases with longer illumination durations. Following the cessation of illumination, the current for the 5 s exposure quickly returns to its initial value. In contrast, the current recovery for the 10 s exposure is relatively slower, and the recovery rate for the 15 s exposure is the slowest. This indicates that by modulating the illumination duration, the recovery time of the current can be effectively extended. This process is also related to the brain’s memory function: the longer the learning duration, the more profound and prolonged the memory effect.

By varying the time interval (Δt) between two UV light stimuli, the device exhibits a paired-pulse facilitation (PPF) effect. This effect is considered one of the most fundamental characteristics in biological synaptic information processing and is crucial for real-time information decoding and transmission. Typically, PPF manifests when two stimuli are applied in quick succession to a presynaptic neuron, resulting in the postsynaptic current from the second stimulus that is greater than that from the first stimulus [43]. This phenomenon is generally attributed to the different rate of neurotransmitter release triggered by the stimuli. Due to the short interval between the two stimuli, the rate of neurotransmitter release triggered by the first stimulus has not fully recovered, thereby resulting in a stronger response to the second stimulus. Figure 3c,d illustrate the PPF effect demonstrated by the device under two brief UV light stimuli. In the experiment, the Δt between two UV light stimuli was set to 5 s, 10 s, 15 s, 20 s, 25 s, and 30 s, respectively. The postsynaptic currents induced by the first and second stimuli are defined as A_1_ and A_2_, respectively, with the PPF index defined as PPF = (A_2_/A_1_) × 100%. As shown in Figure 3c, the PPF decreases as Δt increases, while Figure 3d exhibits the current–time (I–t) curves corresponding to each Δt. The results indicate that the postsynaptic current from the second stimulus is generally larger than that from the first stimulus, with the increment in the second postsynaptic current decreasing as Δt increases. Consequently, the PPF gradually diminishes with increasing Δt. Analogous to biological synapses, when the first UV light stimulus induces an EPSC in the device, a subsequent UV light stimulus applied within a short interval results in a larger EPSC due to insufficient recovery time under dark field conditions. As Δt increases, the degree of current recovery also increases, leading to a decrease in the EPSC induced by the second stimulus. The results indicate that the device exhibits a good PPF effect that decreases with increasing Δt, which are similar to the properties of biological synapses. The device has significant potential for effectively mimicking biological synaptic behavior.

The synaptic plasticity of artificial optoelectronic synaptic devices depends on the persistent photoconductivity (PPC) effect [44,45,46]. PPC refers to the phenomenon where the conductivity of a device does not return to its initial state immediately after the removal of illumination. This is also the reason why the device exhibits STP characteristics. Generally, the photoresponse process of the ZnO thin film is closely related to the adsorption and desorption of oxygen molecules on its surface. Under dark field conditions, oxygen molecules adsorb onto the surface of the film, capturing electrons from within (O_2_ + e^−^ → O^2−^), which leads to the formation of a depletion layer on the surface, thereby reducing the device’s conductivity. When exposed to UV light illumination, photogenerated carriers (hν → h^+^ + e^−^) are generated internally. Photogenerated holes migrate to the surface of the film and recombine with electrons captured by the oxygen molecules (O^2−^ + h^+^ → O_2_), leaving unpaired photogenerated electrons, thereby increasing the device’s conductivity. Upon removal of the UV light illumination, the photogenerated holes recombine with the photogenerated electrons, and the original electrons are recaptured by oxygen molecules, leading to a decrease in the device’s conductivity. The photogenerated holes need to overcome a barrier to return to their initial positions, which is usually slow, resulting in the PPC effect. The PPC effect produced by the ZnO thin film can be fitted with the following functions:

Response process from dark field conditions to UV light illumination:

(1) $$\frac{dI}{dt}=a\left(D-bI\right)\exp\left[c{\left(D-bI\right)}^{2}\right]$$

Response process from UV light illumination to dark field conditions:

(2) $$\frac{dI}{dt}=-a^{\prime }\exp\left[-c{\left(D-b^{\prime }I\right)}^{2}\right]$$

where *D* is the thickness of the ZnO thin film, c is related to the number of ionized donor impurities.

Figure S2 illustrates the switching characteristics of the device. Under UV light illumination, the curve shows an upward trend, whereas under dark field conditions, the curve exhibits a downward trend. We divided the switching process into two phases: the rising edge and the falling edge, and plotted the dI/dt–I curves for each phase separately. Subsequently, we performed functional fitting on the dI/dt–I curves of the rising and falling edges, with the fitting results and experimental data shown in Figure S3a and Figure S3b, respectively. The results indicate that the fitting curves align well with the experimental data.

Based on the above results, the fabricated device exhibits excellent synaptic plasticity. To further enhance the synaptic plasticity, we subjected the device to hydrothermal growth treatment of nanowires, covering its surface with a ZnO nanowire array to increase the specific surface area of ZnO. The detailed processes are as follows: First, the nanowire growth solution was prepared. The growth solution was categorized into two types: one containing ammonia and the other without ammonia. For the ammonia-containing solution, 1.4875 g of Zn (NO_3_)_2_·6 H_2_O (98%, Aladdin, Shanghai, China), 0.3505 g of HMTA (AR, Alfa Aesar, Shanghai, China) and 10.8 mL of ammonia (28%, Alfa Aesar, Shanghai, China) were dissolved in deionized water, bringing the total volume to 200 mL, resulting in a growth solution concentration of 25 mmol/L. The ammonia-free growth solution used the same proportions of raw materials, maintaining a volume of 200 mL and a concentration of 25 mmol/L. Additionally, ammonia-free solutions with concentrations of 50 mmol/L and 100 mmol/L were also prepared. Next, for the ammonia-free group, the prepared devices were placed face down and suspended in the growth solution. The glass bottles containing the growth solution were then heated in an oven at 80 °C for 3 h. For the ammonia-containing group, the growth solution was preheated at 95 °C for 1 h before suspending the device in the solution and then heated at 95 °C for 3 h. Throughout the process, the bottles were kept sealed to prevent air from entering. Finally, the heated devices were removed, rinsed with deionized water, and dried with nitrogen gas, resulting in flexible artificial optoelectronic synaptic devices with ZnO nanowires. We designated the devices prepared with ammonia-free solutions of 25 mmol/L, 50 mmol/L, and 100 mmol/L concentrations as Device 1, Device 2, and Device 3, respectively. The device prepared with the ammonia-containing solution was designated as Device 4.

Figure S4 illustrates the XRD patterns of the devices under different nanowire growth conditions, all showing characteristic peaks corresponding to the ZnO (002) and (004) crystal planes, indicating a c-axis orientation. Figure S5 shows the transmission spectra of the devices, demonstrating low transmittance in the UV region. The PL spectra are illustrated in Figure S6, with the emission peak around 380 nm corresponding to the band-edge emission of ZnO, while the emission peaks in the visible region are attributed to oxygen vacancies within the ZnO.

Figure S7 shows the I–V characteristics of the devices under dark field conditions and varying UV light intensities within a voltage sweep range from −5 V to +5 V, similar to the device without nanowires. Under dark field conditions, the devices exhibit minimal current. Upon exposure to UV light illumination, the current of the devices increases with the intensity of the UV light. Notably, in devices with nanowires, the current in the devices treated with ammonia is of the same order of magnitude as that in the devices without nanowires. In contrast, the current in the devices without ammonia treatment is an order of magnitude higher than that in the devices without nanowires.

**Figure 4:**
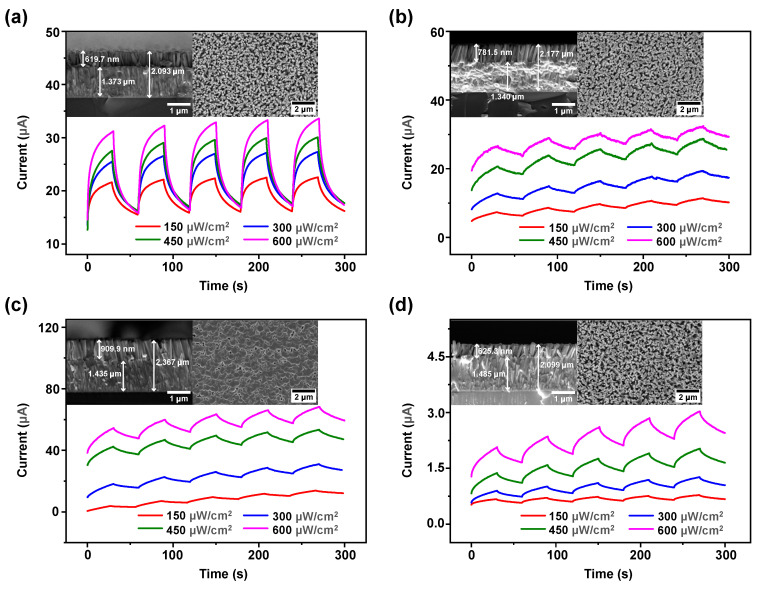
Transient response curves of devices under varying UV light intensities, and SEM images of side view and top view for different nanowire growth conditions. Growth solution concentration of (**a**) 25 mmol/L, ammonia-free; (**b**) 50 mmol/L, no ammonia; (**c**) 100 mmol/L, no ammonia; (**d**) 25 mmol/L, ammonia-containing.

Figure 4 presents the transient response curves of the devices under varying UV light intensities with a fixed +5 V bias, alongside SEM images of the side and top views of ZnO thin films grown with nanowires. The SEM images indicate that the ZnO nanowires are densely packed and uniformly long. Compared to the ZnO thin film without nanowires, the thickness of ZnO thin films with nanowires is slightly reduced, with the nanowires ranging in length from 620 to 910 nm, and the overall ZnO thickness ranging from 2 to 2.4 μm. The transient response curves are similar to that of the device without nanowires. Upon UV light illumination, the devices generate an EPSC. However, after the same duration under dark field conditions, the current does not return to its initial state but remains slightly higher. Device 1 is similar to the device without nanowires, exhibiting STP characteristics. In contrast, Device 2, Device 3, and Device 4 show significantly slower EPSC decay, displaying LTP characteristics, indicating excellent non-volatility in these devices. After five cycles of testing, the EPSC of these three devices progressively increase, further emphasizing the LTP characteristics. In the three groups of nanowires grown without ammonia, the length of the nanowires increased with the concentration of the growth solution, resulting in a higher specific surface area, which is more favorable for light absorption. The growth of the nanowires begins on the seed substrate, and during the initial growth phase, the crystal structure is not fully developed, making it more prone to surface defects. As a result, shorter nanowires have a higher proportion of surface defects, leading to lower charge transport efficiency and carrier lifetime. The addition of ammonia directly introduces OH^−^ into the growth solution, which accelerates the reaction between Zn^2+^ and OH^−^ to form Zn (OH)_2_ [47], promoting the stable growth of ZnO nanowires. This process reduces surface defects, thereby enhancing charge transport efficiency and carrier lifetime. Overall, the EPSC of devices without ammonia treatment is an order of magnitude higher than that of the device without nanowires, with Device 3 exhibiting the highest EPSC. However, Device 1 only exhibits STP characteristics, whereas Device 2 and Device 3 exhibit LTP characteristics. The EPSC of Device 4 with ammonia treatment is of the same order as that of the device without nanowires, but it exhibits LTP characteristics.

As shown in Figure S8, the switching characteristics of devices under varying UV light intensities were tested. With the increase in UV light intensity, both the growth rate and the peak value of the EPSC show an upward trend, indicating that light intensity has a significant impact on the device’s performance. Figure S9 illustrates the response curves exposed to UV light illumination for 5 s, 10 s, and 15 s at the same intensity. The results indicate that as the illumination duration increases, the EPSC shows a gradual increase. Notably, following the cessation of illumination, the recovery rates of the current for different illumination durations are similar. However, due to the different magnitudes of EPSC, their effects differ. Specifically, the current for the 5 s exposure exhibits STP characteristics, the current for the 10 s exposure shows a transforming trend towards LTP characteristics, and the current for the 15 s exposure has the longest recovery time, displaying typical LTP characteristics. By modulating the illumination duration, we successfully achieved the transition from STM to LTM.

**Figure 5:**
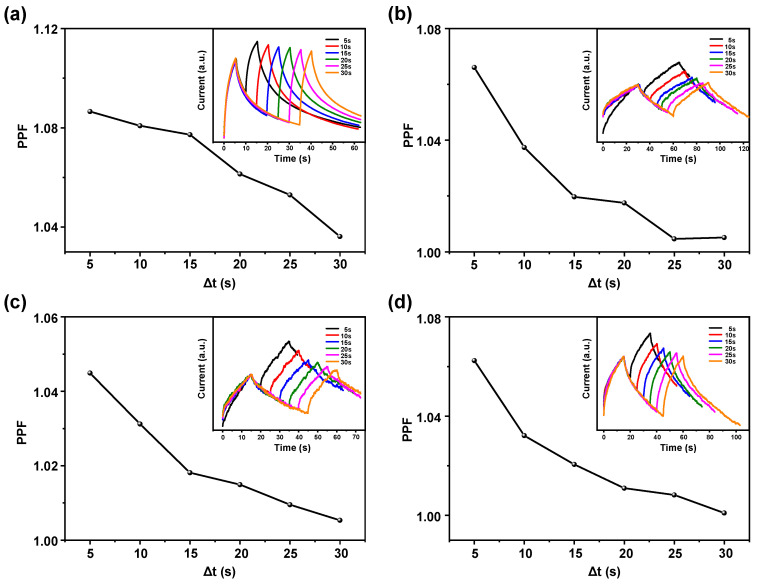
The PPF variation with varying illumination Δt and I–t curves corresponding to each Δt (upper right) for devices under different nanowire growth conditions. Growth solution concentration of (**a**) 25 mmol/L, ammonia-free; (**b**) 50 mmol/L, no ammonia; (**c**) 100 mmol/L, no ammonia; (**d**) 25 mmol/L, ammonia-containing.

Figure 5 illustrates the variation of the PPF of devices with different Δt between two UV light stimuli, alongside the corresponding I–t curves for each Δt. As Δt increases, the PPF gradually decreases, consistent with the typical behavior of biological synapses. It can be observed from the I–t curves that the second postsynaptic current is generally higher than the first. However, as Δt increases, the increment of the second postsynaptic current relative to the first diminished. Consequently, the PPF decreases progressively with the increase in Δt. Notably, at Δt = 5 s, the maximum PPF of the device without nanowires does not exceed 1.01 (Figure 3c), whereas devices with nanowires generally exhibit higher PPF values. This indicates that the nanowires significantly enhance the synaptic plasticity of the devices, suggesting that devices with nanowires have greater potential to mimic biological synapses.

**Figure 6:**
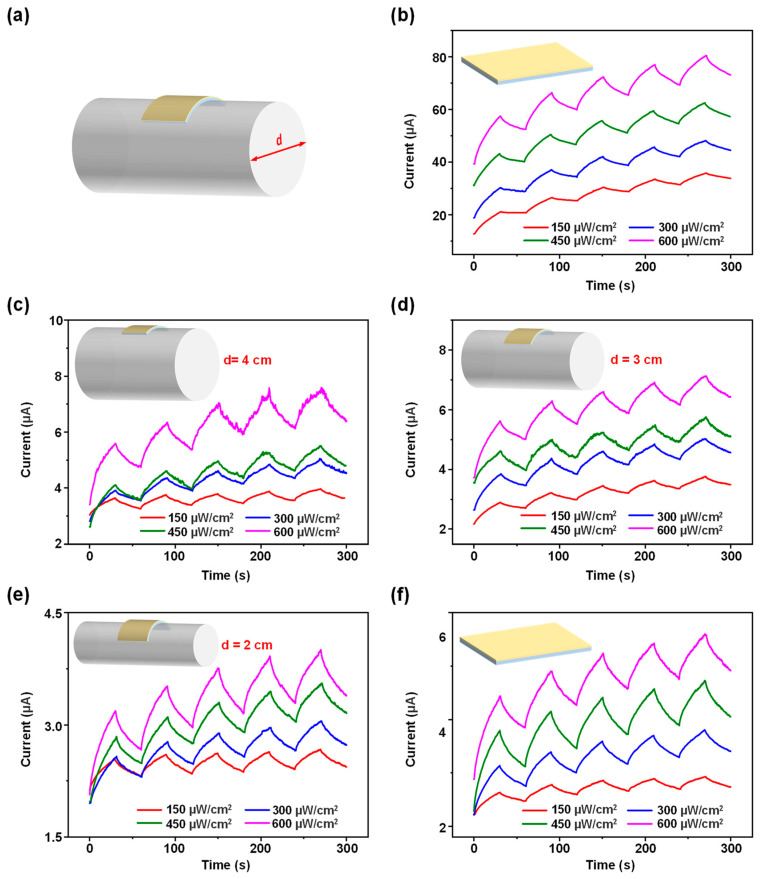
(**a**) Schematic diagram of the bending experiment. Transient response curves under varying UV light intensities for the device with the nanowire growth solution concentration of 100 mmol/L in various bending states. (**b**) Initial state. (**c**) Cylinder rod diameter d = 4 cm. (**d**) Cylinder rod diameter d = 3 cm. (**e**) Cylinder rod diameter d = 2 cm. (**f**) Recovery to the initial state after bending.

To investigate the flexible properties of the device, we conducted bending experiments. As shown in Figure 6a, the backside of Device 3 was placed against an acrylic cylindrical rod to induce bending. Tests were then carried out on the testing platform (Figure 1b). Figure 6b illustrates the transient response curves of the device under varying UV light intensities in an unbent state (initial state), demonstrating current magnitudes on the order of 10^1^, and exhibiting excellent LTP characteristics. Subsequently, Figure 6c–e show the transient response curves as the device was bent around cylindrical rods with diameters of 4 cm, 3 cm, and 2 cm, sequentially, under varying UV light intensities. Finally, Figure 6f presents the transient response curves after the device returned to its initial state, under varying UV light intensities. The results indicate that the current decreases by an order of magnitude when the device is bent. This decrease is attributed to the tensile stress on the ZnO thin film in the bent state, which generates positive charges on the surface, reducing the number of photogenerated holes migrating to the surface and thus decreasing the device’s conductivity, leading to a drop in current. Additionally, after returning to the initial state, the surface smoothness of the ZnO thin film is affected due to the bending, impacting carrier transport efficiency and preventing the current from fully recovering to its initial level. Despite the current reduction in both bent and recovered states, the device consistently maintains stable LTP characteristics, demonstrating excellent synaptic plasticity. These results suggest that the device we fabricated holds excellent potential for applications in flexible neuromorphic computing.

To assess the effect of bending cycles on the device performance, we conducted cyclic bending tests. Transient response curves of the device under different numbers of bending cycles are shown in Figure S11a,b. The current decreases after multiple bending cycles, yet the device retains excellent synaptic plasticity. To quantify the degradation of the synaptic performance, we calculated the relative change in current by measuring the increase in the second postsynaptic current (A_2_) relative to the first postsynaptic current (A_1_), represented as (A_2_ − A_1_)/A_1_, and normalized this value as an indicator of synaptic performance. Figure S10c shows the degradation rate of the synaptic performance across the number of bending cycles. As seen in the figure, the synaptic performance of the device gradually declines with increasing bending cycles, with the performance dropping to 23% of its original value after 100 cycles.

**Figure 7:**
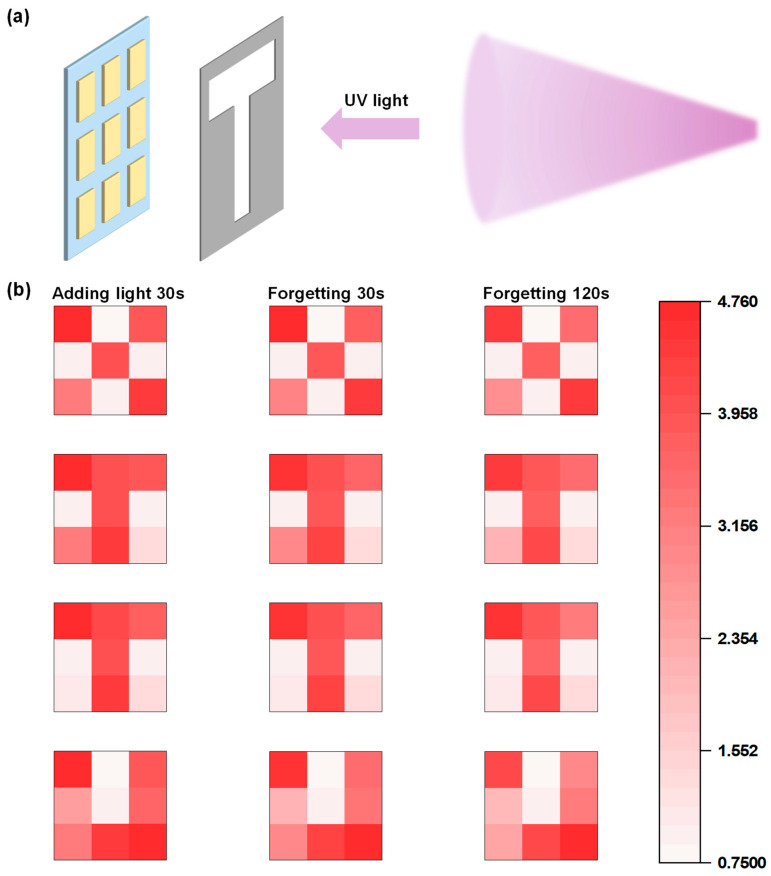
(**a**) The 3 × 3 flexible optoelectronic synapse array and the patterned mask. The UV light irradiates the mask and then illuminate the unshielded devices in the array. (**b**) Images of the array after 30 s of UV light illumination, and following 30 s and 120 s of forgetting. (The color intensity reflects the logarithmic change in device current).

One of the main advantages of optoelectronic synapses is their ability to process optical information. We have designed and fabricated a flexible optoelectronic synapse array with learning and memory functions, where nine devices are arranged in a 3 × 3 grid on a PDMS flexible substrate, as shown in Figure S10. As illustrated in Figure 7a, we employed a patterned mask to block the array, allowing UV light to irradiate the mask and then illuminate the unshielded devices in the array. The devices exposed to UV light exhibit an increase in current, and due to the non-volatile nature of the devices, once the light is removed, the array retains the applied optical information, thus completing the learning-memory process. We used masks with the letters “X”, “J”, “T”, and “U” to shield the array and applied UV light for 30 s, thereby completing the learning process. Afterward, the UV light was removed, and the array was allowed to undergo forgetting under dark-field conditions. Figure 7b shows images of the array’s learned and retained optical information after 30 s of UV light illumination, and the subsequent forgetting processes at 30 s and 120 s, with the color intensity reflecting the logarithmic change in device current. The results indicate that after 30 s of UV light illumination, the array displays the optical information corresponding to the patterns “X”, “J”, “T”, and “U”, and the optical information remains preserved even after 30 s and 120 s of forgetting, demonstrating excellent memory performance.

## 3. Conclusions

In conclusion, we proposed and fabricated a ZnO-based flexible artificial optoelectronic synaptic device. This device exhibits excellent synaptic plasticity, capable of mimicking various biological synaptic functions, including EPSC, STP, and PPF. By improving the fabrication processes, we successfully achieved the growth of nanowires, resulting in a larger specific surface area of ZnO. This improvement further enhances the device’s synaptic plasticity, enabling the exhibition of LTP and the transition from STM to LTM. Moreover, the device demonstrates outstanding flexibility. After bending and recovery, it consistently exhibits stable LTP characteristics. As shown in Table 1, the device in this study is compared with other similar types of devices. While these devices exhibit similar synaptic functions, the device fabricated in this study demonstrates a higher current magnitude, utilize flexible substrates, and feature a simpler fabrication process at a lower cost. This research holds significant promise in the field of artificial intelligence, offering valuable insights for the development of next-generation flexible neuromorphic devices. To overcome current bottlenecks, future efforts should focus on material innovation, exploring novel materials, optimizing packaging technologies, and enhancing the mechanical stability of devices after deformation. Additionally, the manufacturing processes of flexible electronic devices need further refinement to enable low-cost, large-scale production while ensuring consistent performance. In the future, flexible optoelectronic artificial synapses will gradually expand into wearable device applications, such as smart prosthetics, electronic skin, and deeper integration with artificial intelligence and neural networks.

## Figures and Tables

**Table 1:** The device in this work compared with other similar types of device.

Device Structure	Synaptic Functions	Current Magnitude (μA)	Substrate	Reference
ZnO/PDMS	EPSC, STP, LTP, PPF	10^1^	flexible	This work
a-GaO3/ZnO	EPSC, LTP, LTD, PPF	10^1^	flexible	Wang et al. [12]
Ag/AZO/ITO	EPSC, STP, STD, LTP, LTD, PPF	10^0^	rigid	Tang et al. [48]
ZnO/Y7C	EPSC, STP, LTP, PPF	10^−3^	rigid	Song et al. [49]

## Data Availability

The raw data supporting the conclusions of this article will be made available by the authors on request.

## Supplementary Materials

The following supporting information can be downloaded at: https://www.mdpi.com/article/10.3390/s24237788/s1, Figure S1: XRD patterns of the ZnO film; Figure S2: Switching characteristics of the device; Figure S3: dI/dt-I curves based on Figure S2 and the fitting curves. (a) Rising edge. (b) Falling edge; Figure S4: XRD patterns of devices under different nanowire growth conditions. Growth solution concentration of (a) 25 mmol/L, ammonia-free. (b) 50 mmol/L, no ammonia. (c) 100 mmol/L, no ammonia. (d) 25 mmol/L, ammonia-containing; Figure S5: Transmission Spectra of devices under different nanowire growth conditions. Growth solution concentration of (a) 25 mmol/L, ammonia-free. (b) 50 mmol/L, no ammonia. (c) 100 mmol/L, no ammonia. (d) 25 mmol/L, ammonia-containing; Figure S6: PL Spectra of devices under different nanowire growth conditions. Growth solution concentration of (a) 25 mmol/L, ammonia-free. (b) 50 mmol/L, no ammonia. (c) 100 mmol/L, no ammonia. (d) 25 mmol/L, ammonia-containing; Figure S7: I–V characteristics of devices under varying UV light intensities for different nanowire growth conditions. Growth solution concentration of (a) 25 mmol/L, ammonia-free. (b) 50 mmol/L, no ammonia. (c) 100 mmol/L, no ammonia. (d) 25 mmol/L, ammonia-containing; Figure S8: Switching characteristics of devices under varying UV light intensities for different nanowire growth conditions. Growth solution concentration of (a) 25 mmol/L, ammonia-free. (b) 50 mmol/L, no ammonia. (c) 100 mmol/L, no ammonia. (d) 25 mmol/L, ammonia-containing; Figure S9: Transient response curves of devices under different nanowire growth conditions for different illumination durations at the same UV light intensity. Growth solution concentration of (a) 25 mmol/L, ammonia-free. (b) 50 mmol/L, no ammonia. (c) 100 mmol/L, no ammonia. (d) 25 mmol/L, ammonia-containing; Figure S10: Flexible optoelectronic synapse array, where nine devices are arranged in a 3 × 3 grid on a PDMS flexible substrate; Figure S11: Cyclic bending tests. (a) Transient response curves of the device under different numbers of bending cycles (b) Magnified view of the region in (a). (c) Degradation rate of the synaptic performance across the number of bending cycles.
