# Supplementary material for: A Low-Cost Flexible Optoelectronic Synapse Based on ZnO Nanowires for Neuromorphic Computing
Source: Sensors (Basel). 2024 Dec 5;24(23):7788. doi: 10.3390/s24237788 (PMC11645050; doi:10.3390/s24237788)
Supplement: Supplementary file 1 [file sensors-24-07788-s001.zip › sensors-3315634-supplementary.docx]

**Supporting Information**

**A low-cost flexible optoelectronic synapse based on ZnO nanowires for neuromorphic computing**

Yongqing Yue^1,2^, Zixia Yu^1,2^, Fangpei Li^1,2,*^, Wenbo Peng^1,2,*^, Quanzhe Zhu^3^, Yongning He^1,2^

^1^School of Microelectronics, Xi’an Jiaotong University, Xi’an, Shaanxi, 710049, China

^2^The Key Lab of Micro-Nano Electronics and System Integration of Xi’an City, Xi’an, Shaanxi, 710049, China

^3^Shaanxi Advanced Semiconductor Technology Center Co., Ltd., Xi’an 710077, China

^*^Corresponding authors. E-mail: lifangpei@xjtu.edu.cn (Fangpei Li), wpeng33@mail.xjtu.edu.cn (Wenbo Peng)

Figure S1. XRD patterns of the ZnO film.

Figure S2. Switching characteristics of the device.

Figure S3. dI/dt-I curves based on Fig. S2 and the fitting curves. (a) Rising edge. (b) Falling edge.

Figure S4. XRD patterns of devices under different nanowire growth conditions. Growth solution concentration of (a) 25 mmol/L, ammonia-free. (b) 50 mmol/L, no ammonia. (c) 100 mmol/L, no ammonia. (d) 25 mmol/L, ammonia-containing.

Figure S5. Transmission Spectra of devices under different nanowire growth conditions. Growth solution concentration of (a) 25 mmol/L, ammonia-free. (b) 50 mmol/L, no ammonia. (c) 100 mmol/L, no ammonia. (d) 25 mmol/L, ammonia-containing.

Figure S6. PL Spectra of devices under different nanowire growth conditions. Growth solution concentration of (a) 25 mmol/L, ammonia-free. (b) 50 mmol/L, no ammonia. (c) 100 mmol/L, no ammonia. (d) 25 mmol/L, ammonia-containing.

Figure S7. I-V characteristics of devices under varying UV light intensities for different nanowire growth conditions. Growth solution concentration of (a) 25 mmol/L, ammonia-free. (b) 50 mmol/L, no ammonia. (c) 100 mmol/L, no ammonia. (d) 25 mmol/L, ammonia-containing.

Figure S8. Switching characteristics of devices under varying UV light intensities for different nanowire growth conditions. Growth solution concentration of (a) 25 mmol/L, ammonia-free. (b) 50 mmol/L, no ammonia. (c) 100 mmol/L, no ammonia. (d) 25 mmol/L, ammonia-containing.

Figure S9. Transient response curves of devices under different nanowire growth conditions for different illumination durations at the same UV light intensity. Growth solution concentration of (a) 25 mmol/L, ammonia-free. (b) 50 mmol/L, no ammonia. (c) 100 mmol/L, no ammonia. (d) 25 mmol/L, ammonia-containing.

Figure S10. Flexible optoelectronic synapse array, where nine devices are arranged in a 3×3 grid on a PDMS flexible substrate.

Figure S11. Cyclic bending tests. (a) Transient response curves of the device under different numbers of bending cycles (b) Magnified view of the region in (a). (c) Degradation rate of the synaptic performance across the number of bending cycles.
